# Supramolecular Control of the Photoisomerization of a Coumarin-Based Photoswitch

**DOI:** 10.1021/acsomega.4c08106

**Published:** 2024-12-17

**Authors:** Etelka Kiss, Dávid Mester, Márton Bojtár, Zsombor Miskolczy, László Biczók, Dóra Hessz, Mihály Kállay, Miklós Kubinyi

**Affiliations:** † Department of Physical Chemistry and Materials Science, Faculty of Chemical Technology and Biotechnology, Budapest University of Technology and Economics, Műegyetem rkp. 3, 1111 Budapest, Hungary; ‡ MTA-BME Lendület Quantum Chemistry Research Group, Budapest University of Technology and Economics, Műegyetem rkp. 3, 1111 Budapest, Hungary; § ELKH-BME Quantum Chemistry Research Group, Budapest University of Technology and Economics, Műegyetem rkp. 3, 1111 Budapest, Hungary; ∥ Chemical Biology Research Group, Institute of Organic Chemistry, HUN-REN Research Centre for Natural Sciences, Magyar tudósok krt. 2, 1117 Budapest, Hungary; ⊥ Institute of Materials and Environmental Chemistry, Research Centre for Natural Sciences, HUN-REN Research Network, H-1519 Budapest, P.O. Box 286, Hungary

## Abstract

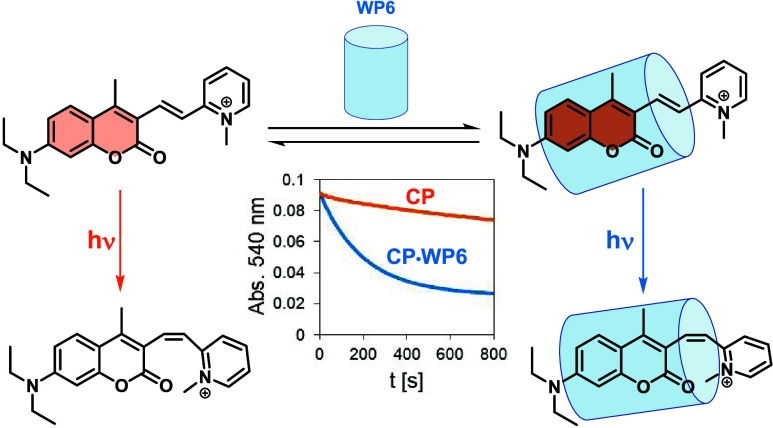

The complex formation of the cationic stilbene-type photoswitch CP with the anionic macrocycles carboxylato-pillar[5]arene (WP5) and carboxylato-pillar[6]arene (WP6) has been investigated in aqueous solution by optical spectroscopic, NMR and isothermal calorimetric experiments and theoretical calculations. Subsequently, the photoisomerization reactions of the supramolecular complexes formed have been studied. CP consists of a 7-diethylamino-coumarin fluorophore and an *N*-methylpyridinium unit, which are connected via an ethene bridge. The *trans* isomer of CP is fluorescent, and its *cis* isomer is dark. The binding constants of the WP6 complexes of the two photoisomers of CP are larger by 2 orders of magnitude than those of the respective complexes with WP5, and *trans*-CP forms more stable complexes with the individual pillararenes than the *cis* isomer. As shown by NMR spectroscopic measurements and theoretical calculations, the two isomers of CP form external complexes with WP5 and inclusion complexes with WP6. On complexation with WP6, the quantum yields of both the *trans*-to-*cis* and *cis*-to-*trans* photoisomerization reactions of CP increase significantly, and the fluorescence quantum yield of *trans*-CP is also enhanced. These changes are due to the suppression of the TICT deactivation process, which is characteristic of 7-dialkylamino-coumarin derivatives.

## 1 Introduction

Molecular switches are small molecules that can be interconverted between two stable or metastable states in response to external stimuli, such as pH, heat, electric field, or light. Among these, light is regarded as a particularly attractive stimulus due to its fast controllability, green applicability and the ability of remote control of chemical processes. Molecular photoswitches are being developed for a wide variety of potential applications, including molecular motors,^1^ photoelectronic devices,^2^ optical information encoding and storage,^3^ solar energy storage,^4,5^ fluorescent biosensors,^6^ photocontrol of biological systems,^7^ photoswitchable catalysis,^8^ super-resolution fluorescence imaging,^3^ and light-activable drugs.^9−11^ Furthermore, photoswitching molecules have great translational potential in retinal vision restoration therapies,^12^ in the suppression of the toxicity of chemotherapeutic drugs^9^ and the side effects of diabetes drugs.^13^

In several photoresponsive systems, supramolecular complexes of molecular photoswitches with macrocyclic hosts are used. The interactions in these complexes are noncovalent, conferring them reversibility, which can be exploited in the construction of smart materials. Examples for such applications are the rotaxane-based molecular muscles, converting UV light into mechanical work^14^ and the molecular motor with a rotaxane-like structure, in which the photoizomerzation of stiff stilbene triggers the nm-scale *trans*lation of a pillararene wheel.^15^ Furthermore, the reversibility of such systems was employed to fabricate photoresponsive sol–gel switching materials,^16,17^ photoactivate the assembly of linear polymers,^18^ convert solid nanoparticles into vesicle-like structures and vice versa,^19−22^ and construct a smart biomimetic nanochannel capable of selective transport of cations and anions.^23^

The differing binding affinity of the host to the two conformational isomers of a photochrome has led to the development of several photoresponsive drug delivery systems. The cucurbituril and sulfonatocalixarene host–guest complexes of the flavylium/*trans*-chalcone photoswitch have been employed in such systems. The light-controlled release of the model drugs was achieved via competitive displacement with the strongly binding flavylium ion.^24,25^ Supramolecular amphiphiles composed of host–guest complexes of cyclodextrins and azobenzene self-assemble into vesicles, which are capable of penetrating the cell membrane.^26^ The vesicular cargo carrier system has the potential to reduce the phototoxicity of photosensitizers used in photodynamic therapy^27^ or to facilitate the delivery of water-insoluble drugs.^28,29^

The applications of the supramolecular complexes of molecular photoswitches in functional materials as well as in diagnostic and therapeutic methods are established by detailed studies of the photochemical and photophysical properties of such complexes in the solution phase. It is anticipated that the rate of the photochromic reaction will be influenced by the spatial confinement of the macrocyclic host. The photochemical reactions of photochromes accompanied by significant conformational change, such as the photoisomerization of azobenzenes,^20,30^ spiropyrans,^31^ and stilbenes,^30,32^ are often inhibited in their host–guest complexes.^31^ In contrast, diarylethenes require minimal conformational freedom, so the increase of the photocyclization quantum yield has been well documented in their cucurbituril complexes.^33,34^ As a specific case, the octaacid complex of an alkyl-azobenzene^35^ also exhibits an increased photoisomerization yield.

The encapsulation of photoswitches within the cavity of macrocyclic hosts can also significantly impact their photophysical properties, which is a particularly important aspect in the case of photoswitchable fluorophores. The complexation can cause blue^20^ and red shifts^24,34^ in their absorption and fluorescence spectra^34,36^ and increase their fluorescence quantum yield^37,38^ and lifetime^33,36,38^ due to the restriction of nonradiative decay processes. Host–guest complex formation in aqueous solutions enhances the resistance to photofatigue^34^ through shielding the guest from water, thereby increasing the number of switching cycles.^33^

Comparative studies on the complexes of photoswitches with the different ring-numbered homologues of the same-type macrocyclic host are particularly instructive, as such studies reveal the trends in the binding affinity and photoreactivity of the complexes formed. Studies on the size-selectivity of cucurbit[*n*]urils^34,39^ and α-, β-, and γ-cyclodextrins^40^ in photoresponsive systems have confirmed that photoisomerization quantum yields increase in accordance with the cavity size in the case of 1:1 complexes. Nevertheless, it is noteworthy that larger cavity-sized hosts also form higher stoichiometry complexes, which can lead to molecular crowding and a subsequent decrease in the reaction rates.

**Figure 1:**
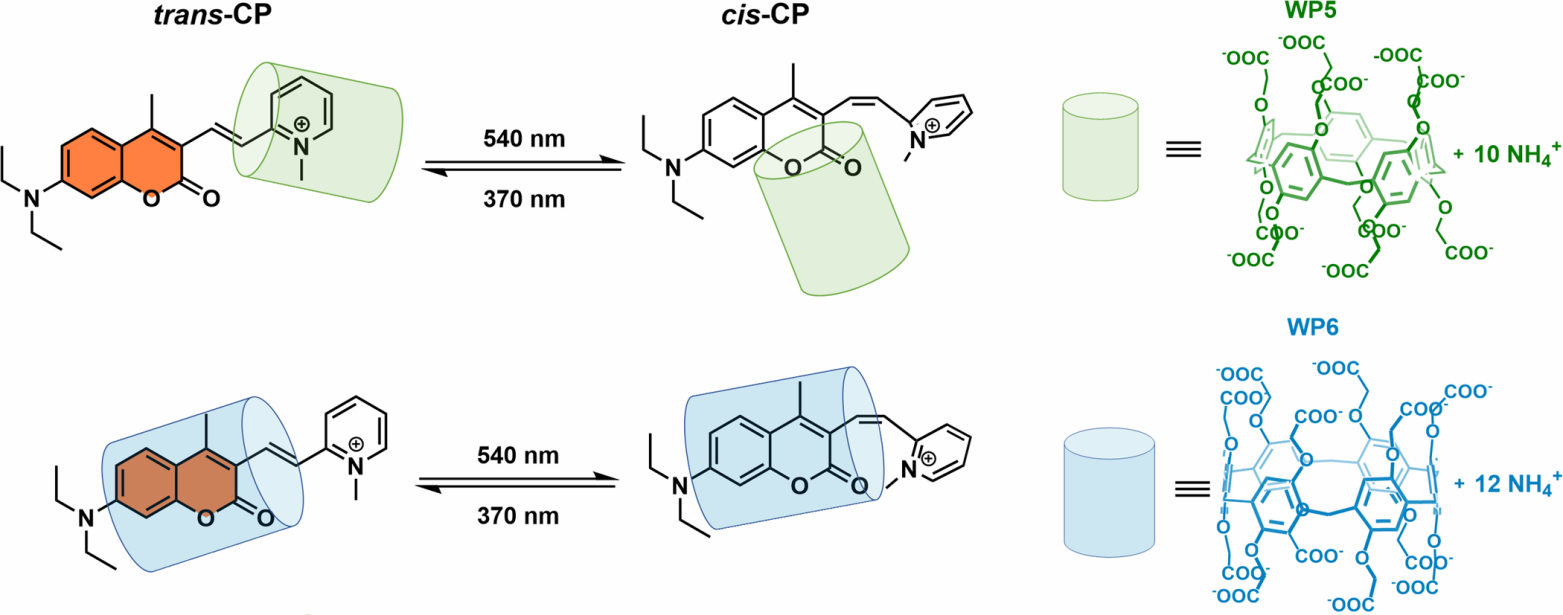
Host–guest complexes of photoswitch CP with pillararenes WP5 and WP6, studied in this work.

In the present work, the structures and the photochemical properties of the complexes formed by the cationic stilbene-type photoswitch CP with the anionic macrocycles carboxylato-pillar[5]arene (WP5) and carboxylato-pillar[6]arene (WP6) were studied (Figure 1). The complexes were characterized by UV–vis, fluorescence and NMR spectroscopic experiments, isothermal titration calorimetry (ITC), and theoretical calculations. The synthesis of CP and its photoswitching characteristics were described in a former paper by our research group.^41^ CP functions as a switchable fluorescent indicator, as its *trans* isomer is fluorescent and its *cis* isomer is dark. Using such fluorophores in microscopic imaging, the interfering autofluorescence of biological samples can be eliminated. One objective of the present study was to explore the impact of complexation on the fluorescence and the photoisomerization of the photoswitch. Although CP has relatively high fluorescence and isomerization quantum yields in acetonitrile, these values decrease in water. Similar to other dialkylamino coumarin dyes, CP also deactivates through a dark TICT state, which is amplified significantly in high polarity solvents like water.^42,43^ A further objective of our study was to probe the size-selectivity of the complexation using two homologue pillararene hosts. To the best of our knowledge, the size-selectivity of pillararenes in host–guest photoswitching systems has not been studied yet.

## 2 Experimental and Theoretical Methods

### 2.1 General

The photophysical and photochemical experiments were carried out in 0.01 M aqueous Tris buffer solution at pH 7.4. At this pH, WP5 and WP6 are present dominantly in their fully dissociated forms.^44,45^ The samples for the UV–vis, fluorescence, and NMR spectroscopic measurements were prepared under dimmed light conditions, and they were kept in the dark until the beginning of the experiments. The NMR spectra were measured on a Varian 500 NMR spectrometer. The UV–vis absorption spectra were recorded on an Agilent 8453 diode array spectrometer. The fluorescence spectra were taken on an Edinburgh Instruments FS5 fluorescence spectrometer. The fluorescence quantum yields were determined using Rhodamine 6G as reference (Φ^F^ = 0.94 in ethanol).^46^ All of the spectroscopic experiments were carried out at 25 °C. Isothermal titration calorimetry (ITC) studies were performed using a MicroCal VP-ITC (GE Healthcare) instrument at 298 K.

### 2.2 Materials

The synthesis of photoswitch *trans*-CP is described in a previous paper of our research group.^41^ WP5 was synthesized by the method of Cragg et al.^47^ For the synthesis of WP6, the method of Huang et al.^19,48^ was used with a minor modification.^49^

### 2.3 Spectroscopic Experiments

The binding constants for the CP·WP5 and CP·WP6 complexes were determined from the absorption, fluorescence, and NMR spectra of mixtures with the same initial CP and varied pillararene concentrations. The fluorescence spectra were corrected for the different absorbances of the samples at the excitation wavelength.

The binding constants of *trans*-CP·WPn 1:1 complexes were determined from the spectral data of the mixtures of the two components containing WPn in excesses. The algorithm was taken from the work of Anslyn et al.^50^*trans*-CP and WP5 also form a 2:1 complex. The method for the determination of the binding constant for the 2:1 stoichiometry complex was taken from the work of Thordarson.^51^ For the determination of the binding constants of *cis*-CP·WPn 1:1 complexes, a photostationary state (PSS) mixture was prepared by UV irradiation of *trans*-CP, and pillararene WPn was added to the PSS mixture. The spectral data of these mixtures were evaluated following the method described by Inoue et al.^52^ The real roots of the cubic equation were determined with the Matlab FZERO function.

### 2.4 Isothermal Calorimetry

The thermodynamics of *trans*-CP complexation with WP5 and WP6 were revealed by an isothermal titration calorimetry method. The samples, prepared in 0.01 M aqueous Tris buffer of pH 7.4, were degassed. In the typical experiments, the cell (volume 1.433 mL) was filled with ≈40 μM titrant solution, and 14 μL volumes of ≈400 μM titrant solution were added stepwise (duration 28 s) from the computer-controlled microsyringe at an interval of 240 s. A stirring speed of 307 rpm was applied to ensure complete mixing. The obtained enthalpograms were corrected by the dilution heats determined in separate experiments under the same condition. The results were analyzed using the two sequential binding steps model of Microcal ORIGIN software. The titrations were repeated at least three times.

### 2.5 Photochemical Experiments

The *trans* → *cis* photoisomerization reactions of the photoswitch CP and its complexes with WP5 and WP6 were studied by irradiating the samples with a 540 nm LED through a 550 nm low-pass filter, whereas the *cis* → *trans* isomerization reactions were induced with a 370 nm LED. The photon fluxes incident on the samples were determined using Ru(bpy)_3_Cl_2_-based actinometry in the visible range^53^ and ferrioxalate actinometry in the near UV range.^54^ The kinetic experiments were carried out in a 1 cm quartz cell, and the photoconversion was monitored by measuring the UV/vis spectra of the samples. The compositions of the photostationary state (PSS) mixtures were determined by ^1^H NMR spectroscopy.

### 2.6 Computational Methods

In the theoretical calculations, the xtb,^55^ crest,^56^ AutoDock Vina,^57^ and Gaussian 09^58^ software packages were used. First, a conformational analysis was performed for both the *trans*- and *cis*-CP molecules at the semiempirical GFN2-xTB level^55^ using the crest algorithm. For the most stable conformers, where the population exceeded 5%, the host–CP complexes were generated using an in-house-developed script and the AutoDock Vina software package. From the obtained structures, several low-energy geometries were selected, covering all possible host–guest orientations. These complexes were further optimized at the wB97X-D/def2-SVP^59,60^ level using the Gaussian program package. As demonstrated, the performance of the applied function is outstanding for ground-state properties,^61^ while the selected basis set provides a reasonable compromise between accuracy and computational requirements. To mimic the experimental conditions, all semiempirical and density functional theory calculations were performed using water as the solvent.^55,62,63^

## 3 Results and Discussion

### 3.1 Binding of the Photoisomers of CP by Pillararenes

**Figure 2:**
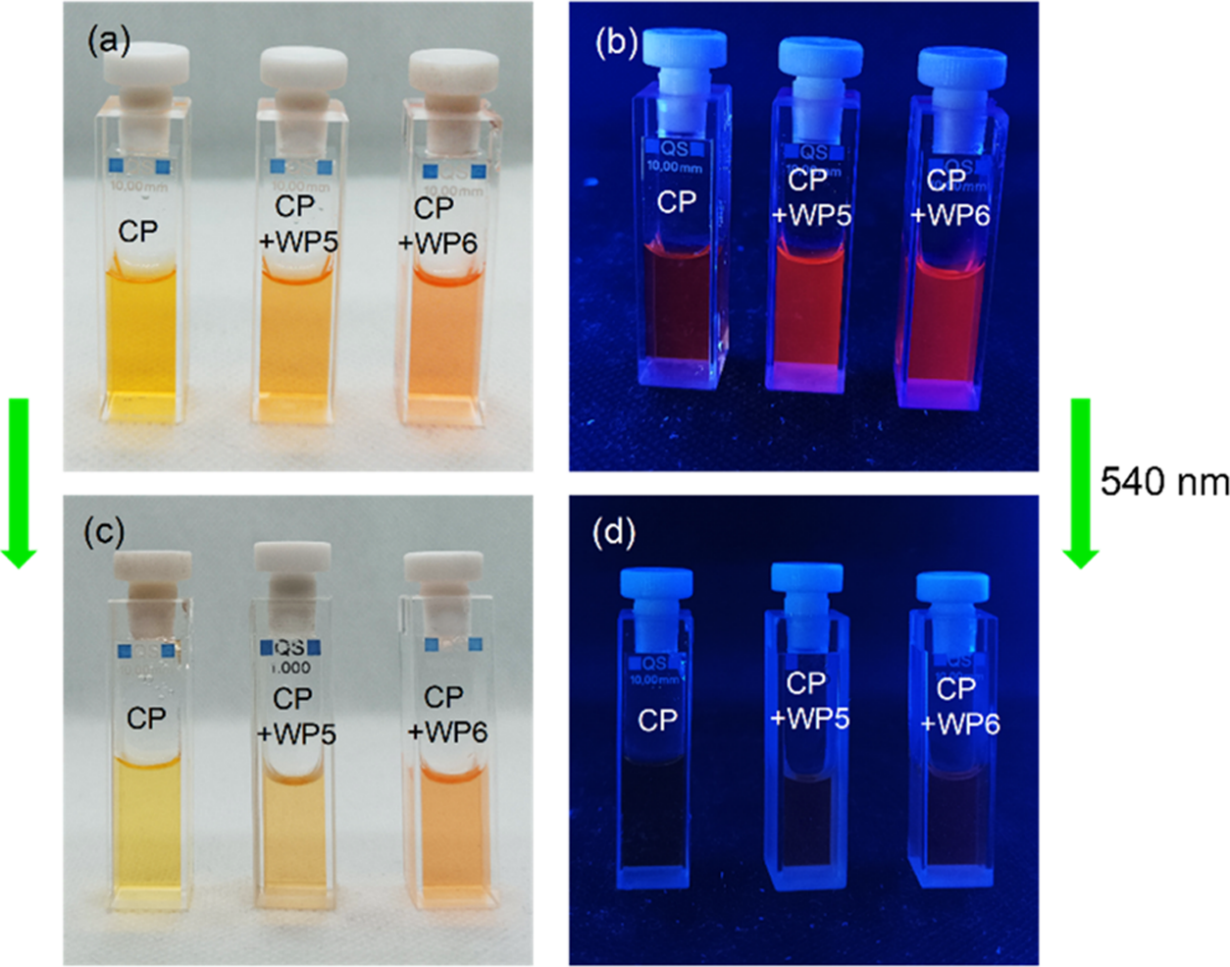
Photographs of a 2 × 10^–5^ M solution of *trans*-CP and its mixtures with 32 equiv of WP5 and 16 equiv of WP6 taken under (a) visible and (b) UV light; (c, d) photographs of the same samples after irradiating them with a 540 nm LED for 70 s. The solvent is Tris buffer (pH = 7.4).

It was found in a preliminary experiment that the yellow solution of *trans*-CP turned to orange on the addition of WP5 and WP6 in excess, and the weakly fluorescent solutions became strongly fluorescent (see Figure 2). These effects were more pronounced for the CP–WP6 mixture. After the irradiation of the solutions with the green (540 nm) LED, the colors of the samples were faded, and their fluorescence was quenched.

**Figure 3:**
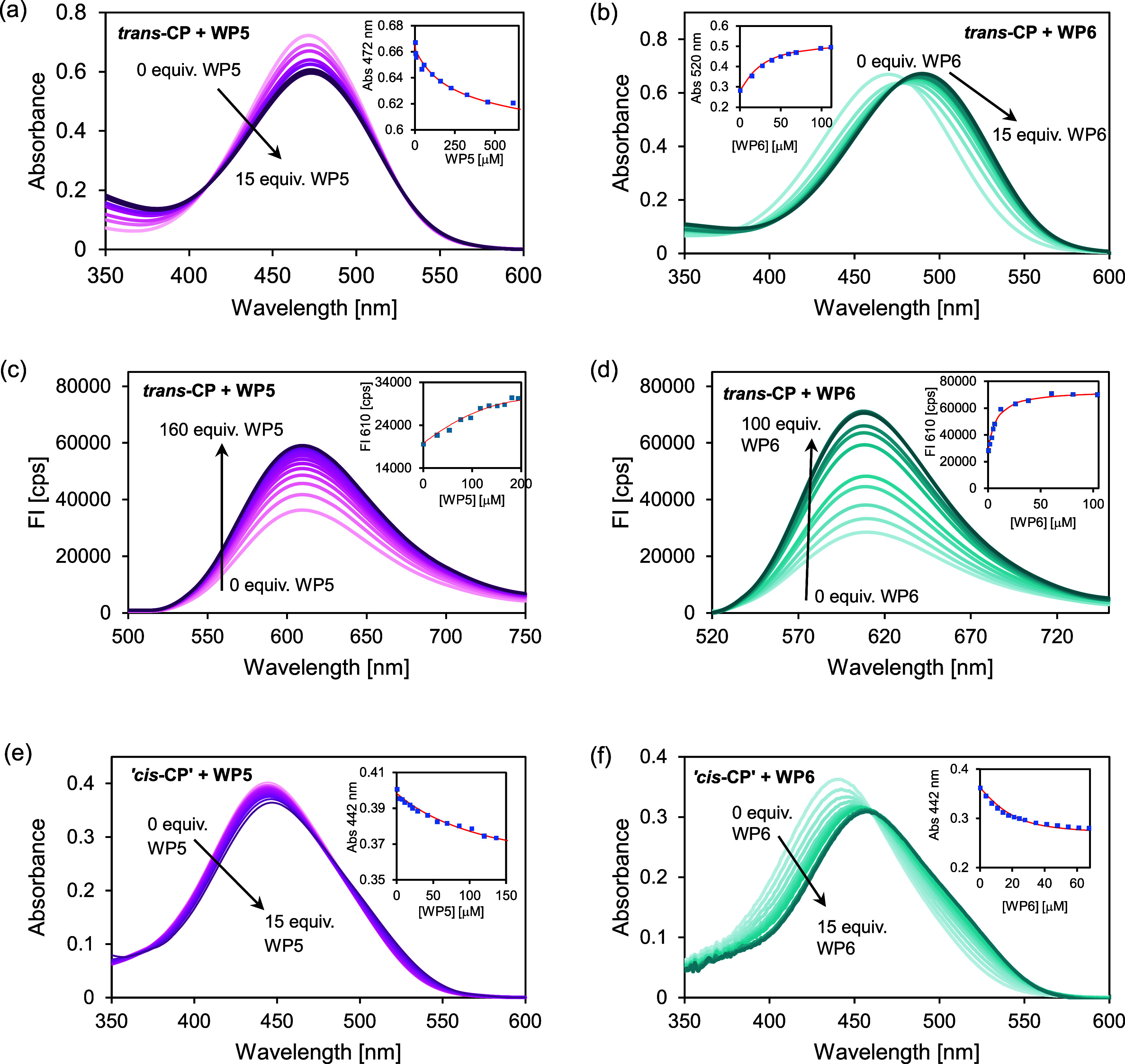
(a–d) Absorption and fluorescence spectra of *trans*-CP and (e, f) absorption spectra of a PSS mixture with 75% *cis*-CP in the presence of WP5 and WP6 in different concentrations. The initial concentrations of the photoswitch are (a, c) [*trans*-CP]_0_ = 2 × 10^–5^ M, (b, d) [*trans*-CP]_0_ = 2 × 10^–6^ M, and (e, f) [*trans*-CP + *cis*-CP]_0_ = 2 × 10^–5^ M. The fluorescence spectra were recorded with excitation at 500 nm. The insets show the measured and nonlinear fitted intensities or absorbances at a selected wavelength vs. the concentration of pillararene.

The absorption and fluorescence spectra of *trans*-CP in the presence of WP5 and WP6 in different concentrations are shown in Figure 3a–d. As can be seen, the absorption band shows only minor changes on the addition of WP5, whereas it shows a distinct redshift on the addition of WP6. The position of the fluorescence band of *trans*-CP remains unchanged in the presence of the two pillararenes but the intensity of the band increases. The fluorescence quantum yield of *trans*-CP is Φ^F^ = 0.011 in aqueous solution, and this value increases to Φ^F^ = 0.024 at high WP5 excesses and to Φ^F^ = 0.027 at high WP6 concentrations.

As the *cis* isomer of CP is nonfluorescent and it could not be obtained in pure form, its complexation was studied by measuring the absorption spectra of a PSS mixture with *cis*-CP excess in the presence of WP5 and WP6 in different concentrations. These spectra can be seen in Figure 3e,f where “*ci*s-CP” is in fact a PSS mixture.

The binding constants of the photoswitch-pillararene complexes, which exhibited different magnitudes, were determined from the absorption and fluorescence spectra. In addition, NMR and isothermal calorimetric experiments were also carried out for this purpose (see Sections 3.2 and 3.4).

The pillararene hosts WP5 and WP6 with their two portals can form 1:1 as well as 2:1 complexes with appropriate guest molecules.^44,64^ In the samples used in the absorption and fluorescence spectroscopic experiments, in which the pillararene hosts were in excess, the formation of only 1:1 complexes was observed. Simultaneous occurrence of 2:1 and 1:1 complexes was indicated by the calorimetric experiments and the NMR spectra.

The binding constants for the complexes of the *trans* isomer
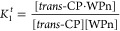
1were determined first, by nonlinear fittings to the absorption and fluorescence spectra of the *trans*-CP – pillararene mixtures. The binding constants for the *cis* isomer
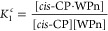
2were obtained then from the absorption spectra of the *cis*-CP – *trans*-CP PSS mixtures containing WP5 or WP6 in different concentrations, considering the formation of two 1:1 complexes, *cis*-CP·WPn and *trans*-CP·WPn in these systems, with binding constants, *K*_1_^*c*^ and *K*_1_^*t*^. As the value of *K*_1_^*t*^ was determined previously, the value of *K*_1_^*c*^ could be obtained by a single parameter nonlinear fitting to the spectra.

The occurrence of 2:1 (*trans*-CP)_2_·WP5 and 2:1 (*trans*-CP)_2_·WP6 complexes was suggested by the NMR spectra and calorimetric data of samples with *trans*-CP excesses. Their binding constants were written in the form
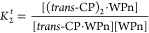
3

The binding constants obtained by different methods are listed in Table 1. The respective *K*_1_^*t*^ values obtained by the different experimental methods are in reasonable agreement. As can be seen, both the *trans* and the *cis* isomers of CP bind much stronger to WP6 than to WP5, demonstrating that these complexations are strongly size-selective reactions. Furthermore, comparing the binding constants of the two isomers with the same pillararenes, the value for *trans*-CP·WP5 is *ca*. four times higher than for *cis*-CP·WP5, whereas the difference between the binding constants for *trans*-CP·WP6 and *cis*-CP·WP6 is even lower, which is below the experimental error. Thus, in these systems, the capture and release of the photoswitch guest cannot be controlled efficiently by irradiation with UV and visible light.

**Table 1:** Binding Constants of CP·WP6 and CP·WP5 Complexes

complex	*K* [M^–1^]	method of determination
*trans*-CP·WP5	(4.3 ± 1) × 10^3^	absorption spectroscopy
	(2.77 ± 0.05) × 10^3^	fluorescence spectroscopy
	(3.2 ± 0.3) × 10^3^	NMR
(*trans*-CP)_2_·WP5	(8 ± 3) × 10^2^	NMR
*cis*-CP·WP5	(1.4 ± 0.8) × 10^3^	absorption spectroscopy
*trans*-CP·WP6	(1.37 ± 0.18) × 10^5^	absorption spectroscopy
	(1.45 ± 0.07) × 10^5^	fluorescence spectroscopy
	(1.6 ± 0.2) × 10^5^	isothermal calorimetry
(*trans*-CP)_2_·WP6	(9.2 ± 2) × 10^3^	isothermal calorimetry
*cis*-CP·WP6	(1.31 ± 0.18) × 10^5^	absorption spectroscopy

It is to be noted that like in most of the studies on supramolecular complexes, the binding constants in Table 1 are based on concentrations, and the activity coefficients of the solutes are disregarded. In the samples used in the NMR experiments, the concentrations of the ionic hosts and guests were relatively high; thus, due to the electrostatic interactions between the ionic species, the activity coefficients could differ noticeably from unity.

The errors of the binding constants of host–guest complexes, determined by spectroscopic methods, depend on the errors of concentrations, the instrumental noise, and the overlap of the spectral bands of the free and complexed guest.^65^ The large error in the case of *cis*-CP·WP5 is due to the large overlaps of the absorption bands of the free *cis* isomer and its WP5 complex. As can be seen in Figure 3, the addition of WP5 to *cis*-CP causes only a slight change in the absorption spectrum. The relatively high errors of the binding constants of the 2:1 complexes arise primarily from the low concentrations of these species in the samples studied, where the 1:1 complexes dominated over the 2:1 complexes, in accordance with the differences in the respective binding constants.

### 3.2 Isothermal Calorimetry

**Figure 4:**
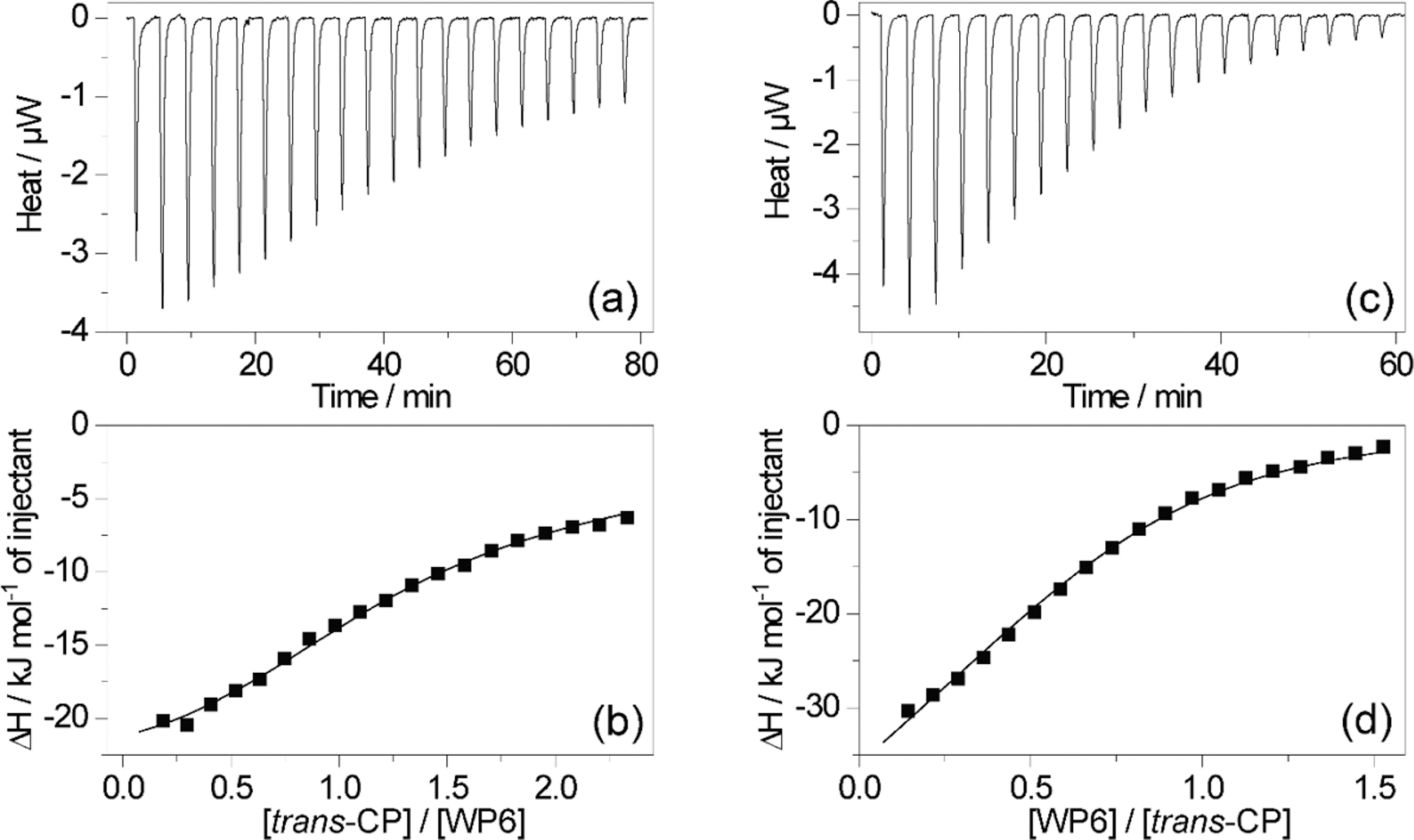
(a, c) Results of isothermal calorimetric titrations. (b, d) Integrated heat released per addition divided by the concentration of the injectant (■) was plotted as a function of the molar ratio of the reactants for the titration of (a, b) 37 μM WP6 with 410 μM CP and (c, d) 41 μM CP with 420 μM WP6 in 0.01 M aqueous Tris buffer at pH 7.4 at 298 K.

The inclusion of the *trans* isomer of CP in WP6 was also studied by isothermal calorimetric titrations to gain insight into the driving force of association. Two types of measurements were carried out. Figure 4a,b displays the results obtained by the gradual addition of 410 μM *trans*-CP to 37 μM WP6 solution, whereas the data collected by the successive injections of 420 μM WP6 aliquots to a 41 μM *trans*-CP solution are presented in Figure 4c,d. The enthalpograms did not obey the reversible 1:1 association model, but the experimental data could be fitted well, assuming sequential binding of two *trans*-CP guests to WP6. The global analysis of the results of the two measurement types provided the binding constants listed in Table 1 and the thermodynamic data summarized in Table 2. The binding constant of 1:1 complexation (*K*_1_^*t*^) attained by isothermal titration calorimetry (ITC) agreed well with those acquired by absorption and fluorescence spectroscopic methods. In the case of the latter techniques, a much smaller *trans*-CP concentration was employed, which prevented the association of *trans*-CP with the *trans*-CP·WPn complex allowing thereby the selective *K*_1_^*t*^ determination. The lower sensitivity of the ITC method required the use of larger reactant concentrations where 2:1 complexation had a substantial contribution. The binding constant of this process, *K*_2_^*t*^, was found to be much lower than *K*_1_^*t*^ (Table 1), indicating the negative cooperativity for the second *trans*-CP confinement. Table 2 includes the Gibbs free energy changes in the first (Δ*G*_1_^*t*^) and second (Δ*G*_2_^*t*^) complexation steps calculated by the following relationship: Δ*G*_*n*_^*t*^ = −*RT* ln *K*_*n*_^*t*^. Although Δ*G*_2_^*t*^ is less negative than Δ*G*_1_^*t*^, much more heat evolves in the second host–guest binding equilibrium. The 1:1 self-assembly is accompanied by a considerable entropy gain, while the incorporation of the second *trans*-CP is highly unfavorable. Similar phenomena were found for alkaloid inclusion in cucurbit[8]uril macrocycle.^66,67^ Biederman and co-workers demonstrated that the release of high-energy water from the interior of cavitands is the essential enthalpic driving force for host–guest complexation.^68,69^ Like in other hosts, water molecules have higher energy in the hydrophobic WP6 core than in the bulk solution because they interact weaker and do not form an optimized hydrogen-bond network. The 1:1 inclusion complex formation leads to the partial release of the high-energy water, causing enthalpy diminution. However, the energetic frustration of the remnant water grows in the small space left after entry of the first guest in WP6. Therefore, a larger enthalpy gain is obtained when a second guest expels these water molecules. Table 2 shows that both the 1:1 and 2:1 encapsulations of *trans*-CP in WP6 are exothermic, enthalpy-controlled processes. Substantial entropy gain contributes to the driving force of 1:1 complexation. This implies that the entropy loss originating from the loose host–guest association and the integration of the water molecules displaced from the host cavity into the hydrogen-bonded network in the bulk are overbalanced by the entropy increase due to the desolvation of the reactants. In contrast, the embedding of the second *trans*-CP into *trans*-CP·WP6 results in a highly unfavorable entropy change because the degrees of freedom of the constituents are substantially limited in the produced tightly packed ternary complex. In addition, the large conformational freedom of the high-energy water within the partially filled WP6 cavity is reduced when the penetration of the second *trans*-CP forces these water molecules to join the solvent network in the bulk.

**Table 2:** Thermodynamic Data for the Consecutive Bindings of *tran*s-CP Guests to a WP6 Host

Δ*G*_1_^t^	–29.7 ± 0.3 kJ mol^–1^Δ*G*_1_^*t*^)
Δ*H*_1_^*t*^	–24.8 ± 0.2 kJ mol^–1^
Δ*S*_1_^*t*^	16 ± 2 J mol^–1^ K^–1^
Δ*G*_2_^*t*^	–22.6 ± 0.6 kJ mol^–1^
Δ*H*_1_^*t*^	–42 ± 5 kJ mol^–1^
Δ*S*_1_^*t*^	–65 ± 17 J mol^–1^ K^–1^

In similar experiments on the *trans*-CP–WP5 system, only a small heat evolution was observed. It was commeasurable with the heat of dilution of the WP5 titrant solution, preventing the determination of the thermodynamic data but confirming that *trans*-CP forms a much weaker complex with WP5 than with WP6 in a very weak exotherm process. The low binding affinity to WP5 probably arises primarily from steric reasons. Moreover, molecular-dynamics simulations showed that the size of the unsubstituted pillar[5]arene is too small to contain a significant amount of water.^69^ Therefore, the release of high-energy water cannot contribute to the stability of its complexes.

### 3.3 Photoisomerization Studies

#### 3.3.1 Kinetic Equations

The quantum yields of the photoisomerization reactions of the photoswitch CP in the free state and in its complexes with the pillararenes, Φ_CP_^*t*→*c*^, Φ_CP_^*c*→*t*^, Φ_CP·H_^*t*→*c*^, and Φ_CP·H_^*c*→*t*^, were determined from the initial slopes of the time-dependent absorbance data of four samples, irradiated by the green or the UV LED.

Φ_CP_^*t*→*c*^ was obtained from the absorbance data of a sample containing initially only the pure *trans* isomer, which was irradiated by the green LED. The initial rate of the photochemical reaction in this sample is
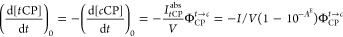
4where *I*_*tCP*_^abs^ is the light flux absorbed by the *trans* isomer of the photoswitch, *I* is the incident light flux, (photon fluxes in mole photon s^–1^), *V* is the volume of the sample, and *A*^E^ is the absorbance at the wavelength of irradiation, λ^E^. The initial rate of the absorbance change at the detection wavelength λ^D^, arising from the consumption of the *trans* isomer and the formation of the *cis* isomer, is
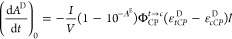
5where ε_*tCP*_^D^ and ε_*cCP*_^D^ are the absorption coefficients of the two isomers at λ^D^, and  is the optical path length.

The samples used for the determination of the other photoisomerization quantum yields contained two or more species absorbing at λ^E^ even at the start of the irradiation. In such systems, the light flux absorbed by the *i*-th component is^70,71^ (*i* = *tCP, cCP, tCP·H, cCP·H*)
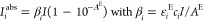
6Φ_*CP·H*_^*t* → *c*^ was determined from the absorbance data of a sample, which was initially a mixture of the *trans* isomer, the respective pillararene host, and their host–guest complex. This sample was also irradiated with the 540 nm LED. In this case, the initial slope of the *A*^D^(*t*) function consists of two terms, corresponding to the *trans*-to-*cis* photoisomerization of the free guest and its complex
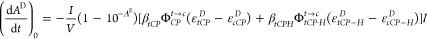
7

As the pure *cis* form of the photoswitch was not available, for the determination of Φ_*CP*_^*c* → *t*^, a PSS mixture was used in which the *cis* isomer was in excess, and it was irradiated by the UV LED. For such a system, both the *cis*-to-*trans* and the *trans*-to-*cis* photoisomerizations of the photoswitch contribute to the spectral changes even at the initial stage of the irradiation. Therefore, the slope of the initial tangent for the measured *A*^D^(*t*) curve has the form
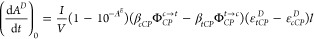
8

Finally, Φ_*CP·H*_^*c* → *t*^ was determined from the *A*^D^(*t*) data of the above PSS mixture to which the respective pillarane was added, and the system was irradiated then with the UV LED. Taking into account that for this system, all four photochemical reactions—the *trans*-to-*cis* isomerization of the free photoswitch and its complex and the reverse processes—affect the *A*^D^(*t*) curve even at the start of the irradiation
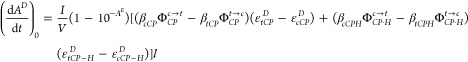
9

#### 3.3.2 Photokinetic Experiments

The photon fluxes of the LED lights, incident to the samples, *I*, were determined by actinometry. Their values were *I* = 4.02 × 10^–9^ mole photon s^–1^ for the green LED and 2.64 × 10^–9^ mole photon s^–1^ for the UV LED. The total concentration of the photoswitch was 2 × 10^–5^ M in each experiment. In the samples used for the determination of Φ_*CP·H*_^*t* → *c*^ and Φ_*CP·H*_^*c* → *t*^, the pillararene hosts were added in excesses, and their total concentrations were [WP5]_0_ = 5.65 × 10^–4^ M and [WP6]_0_ = 1.91 × 10^–4^ M, in order to make the contributions of the complexed photoswitch dominant to the signal. For the calculations of the β_*i*_ fractional absorbances in the photoswitch–pillararene mixtures according to eq 6, the initial concentrations of the pure and complexed photoswitch isomers were obtained from the association constants in *K*_1_^*t*^ and *K*_1_^*c*^ in Table 1.

**Figure 5:**
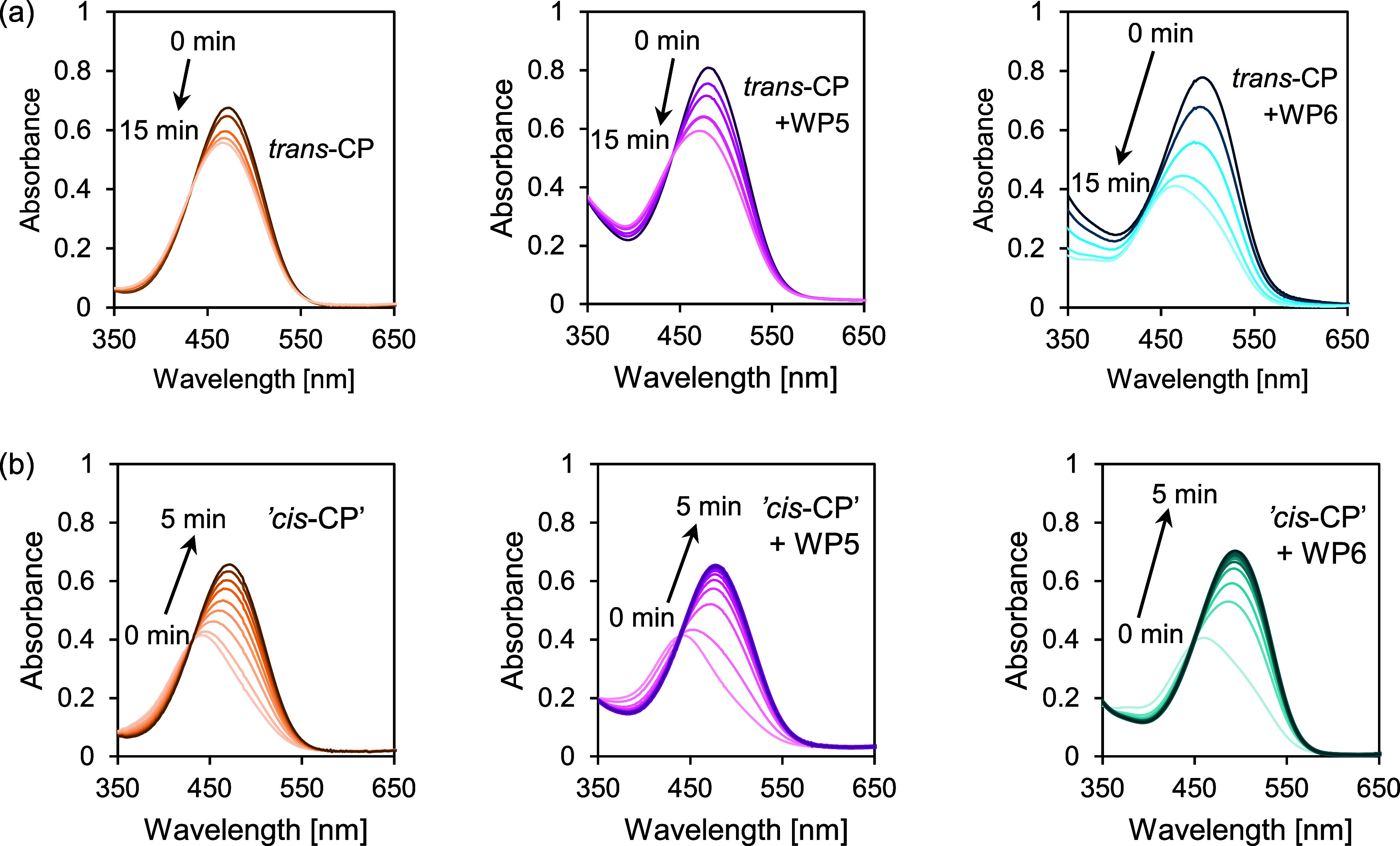
(a) Variations of the absorption spectra of *trans*-C*P* and its mixtures with WP5 and WP6 on irradiation by a green (540 nm) LED. (b) Variations of the absorption spectra of *trans*-CP/*cis*-CP PSS with 75% *cis* isomer containing no pillararenes and containing WP5 and WP6, on irradiation with an UV (370 nm) LED. Initial concentrations: [CP]_0_ = 2 × 10^–5^ M in each solution, [WP5]_0_ = 5.65 × 10^–4^ M, [WP6]_0_ = 1.91 × 10^–4^ M.

The evolution of the absorption spectra of the solutions of the pure *trans* photoswitch and its mixtures with WP5 and WP6, irradiated by the green LED, as well as the variation of the spectra of the samples “*cis*-CP”, “*cis*-CP” + WP5, and “*cis*-CP” + WP6 under irradiation by the UV LED are shown in Figure 5 (“*cis*-CP” denotes the same PSS mixture as in Figure 3). It can be clearly seen in the figures that both the *trans*-to-*cis* and *cis*-to-*trans* photoisomerization reactions of the complexed photoswitch are faster than the respective reactions of the free molecule.

The absorbance values of the samples at λ^D^ = 540 nm, measured at early times of the experiments, are plotted in Figure S3. As can be seen, the *A*^D^(*t*) functions in these time intervals can be considered linear with a good approximation.

The photoisomerization quantum yields calculated from the slopes of the lines fitted to the initial *A*^D^(*t*) data are collected in Table 3. As indicated by the figures in the table, the complexation of CP by WP6 results in a significant (≈2-fold) increase of the quantum yields of both the *trans*-to-*cis* and the *cis*-to-*trans* photoisomerizations. The complex formation with WP5 also enhances the efficiencies of the photoreactions but to a lower extent.

**Table 3:** Quantum Yields and PSS Compositions for the Photoisomerization Reactions of Photoswitch CP and Its Complexes with WP6 and WP5

compound	Φ^*t*→*c*^	PSS^*t*→*c*^	Φ^*c*→*t*^	PSS^*c*→*t*^
CP	0.046	75% *cis*	0.25	99% *trans*
CP·WP5	0.056	66% *cis*	0.28	98% *trans*
CP·WP6	0.090	63% *cis*	0.47	98% *trans*

**Figure 6:**
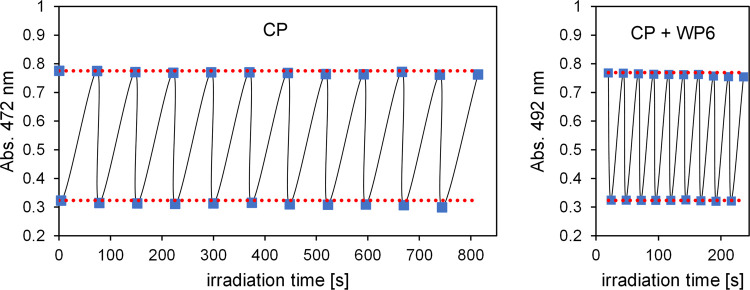
Photoswitching of CP and a CP–WP6 mixture induced by alternating visible (540 nm, 70 s) and UV (370 nm, 4 s) irradiations. The concentration of CP is 2 × 10^–5^ M in both samples, and the CP–WP6 mixture contains 10 equiv. WP6. The absorbances are measured at the band maxima.

The enhancement of the photoisomerization quantum yields in the presence of WP6 results in a faster operation of the photoswitch. This is demonstrated in Figure 6, and the switching between two states of similar absorbance values is achieved four times faster using the CP–WP6 system than when using pure CP.

### 3.4 NMR Spectra

A further insight into the complexation modes of the photoisomers of the photoswitch CP with the pillararenes WP5 and WP6 has been obtained by ^1^H NMR spectroscopy. As the components and their complexes are readily soluble in water, the spectra were measured in D_2_O. First, the NMR spectra of *trans*-CP were measured in the absence of pillararenes and in the presence of WP5 or WP6 at different concentrations, and the displacements of the NMR signals were followed. Then, the solutions of pure *trans*-CP and its 1:1 mixtures with WP5 and WP6 were converted to PSS mixtures by irradiation by the 540 nm LED, and the spectra of the PSS mixtures were taken.

**Figure 7:**
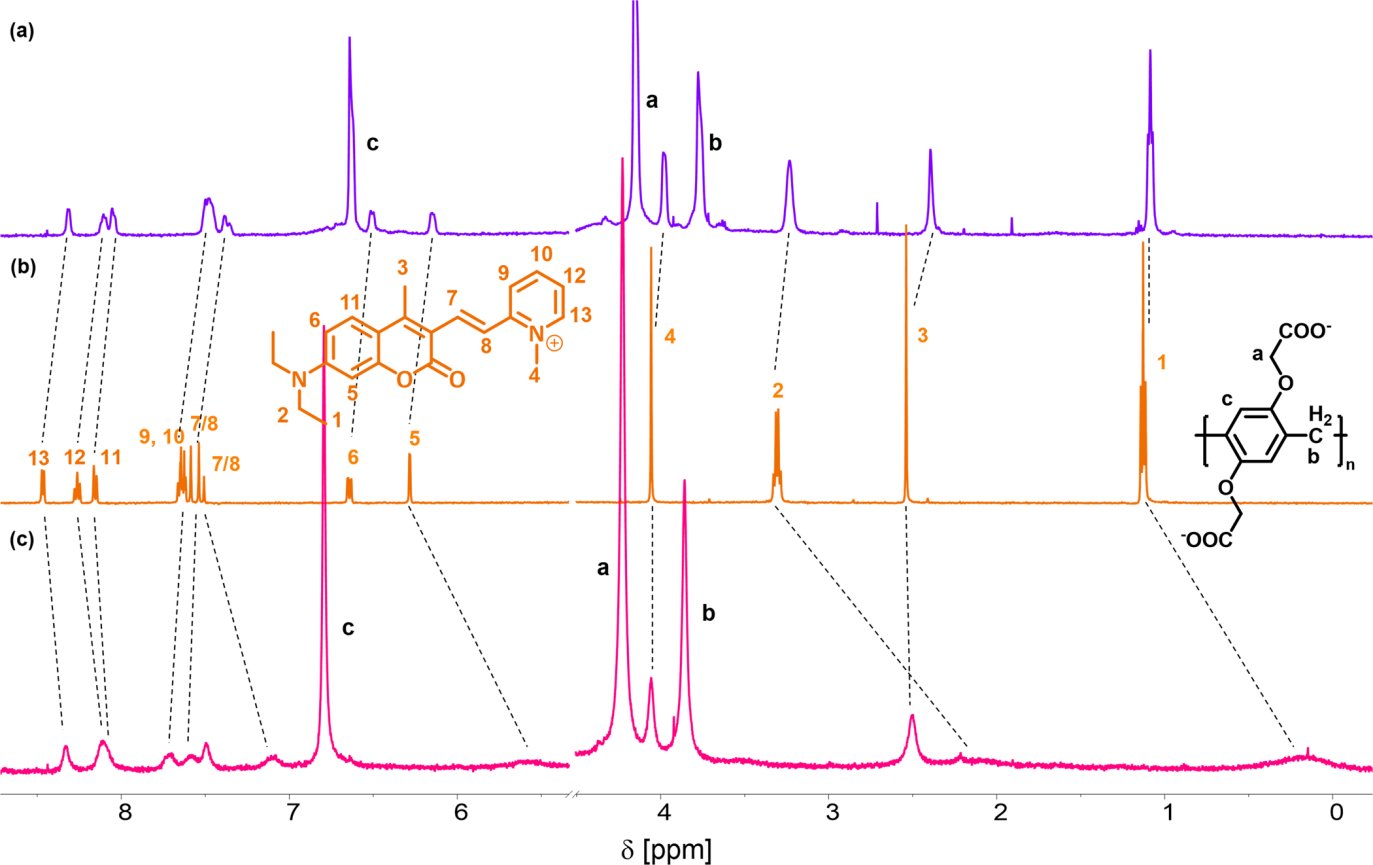
^1^H NMR spectrum of (a) *trans*-CP and 1 equiv of WP5, (b) *trans*-CP, and (c) *trans*-CP and 1 equiv of WP6 at 500 MHz, D_2_O; [CP] = 6 × 10^–4^ M.

The spectra obtained from the 1:1 *trans*-CP–WP5 and *trans*-CP–WP6 mixtures are illustrated in Figure 7. On the addition of both pillararene hosts, most of the signals of the guest undergo an upfield shift. In the spectrum of the 1:1 mixture of *trans*-CP with WP5, the signals of the pyridinium moiety are shifted slightly more than the signals belonging to the diethylamino group, suggesting that the pyridinium moiety of the photowitch intrudes into the cavity of the pillararene host. In contrast, in the spectrum obtained from the *trans*-CP–WP6 mixture, the signals of the protons on the diethylamino side of *trans*-CP shift significantly upfield, whereas the signals belonging to the pyridinium group remain almost unchanged. This indicates a structure in which the diethylamino side of the *trans*-CP is located largely within the cavity of the WP6 host.

The spectra of the same samples, after irradiating them with the green LED for 10 min, are shown in Figure S4. From a solution of *trans*-CP, a PSS mixture with 75% *cis*-CP was obtained, as indicated by the relative intensities of the NMR signals. The irradiation of the *trans*-CP WP5 equimolar mixture for the same duration provided a sample with 55% (complexed) *cis*-CP. Comparing the spectra of these two samples (Figure S4a,b), it can be seen that—like the signals of the *trans* isomeric photoswitch—the signals of *cis*-CP also shift upfield on complexation. Most of these shifts, however, are smaller than the complexation-induced shifts of the signals of *trans*-CP, except for the signals of the diethylamino protons, which display larger shifts in the spectrum of the complexed *cis* isomer. These differences indicate different binding modes in the WP5 complexes of the *cis* and *trans* forms of CP.

In the spectrum measured after irradiating the *trans*-CP–WP6 mixture with the green LED (see Figure S4c), the complexation-induced shifts of *cis*-CP and *trans*-CP are of comparable extent. The signals of the diethylamino protons of *cis*-CP exhibit substantial upfield shifts, while the signals of its pyridinium group exhibit only minor shifts, suggesting that like *trans*-CP·WP6, *cis*-CP·WP6 is also an internal complex, with the diethylamino-coumarin moiety of the guest enclosed in the macrocyclic cavity.

A more complete set of spectra recorded on samples at constant *trans*-CP and various WP5 concentrations is presented in Figure S5. It can be clearly seen that at low [WP5]/[*trans*-CP] ratios, the signals of both components shift upfield rapidly with growing WP5 concentrations, and at about a ratio of [WP5]/[*trans*-CP] = 0.5, this trend reverses and the upfield shifts of the signals diminish. Such variation of the spectra suggests that in addition to the 1:1 complex, *trans*-CP also forms a 2:1 complex with WP5. The 2:1 complex can be detected at low WP5 concentrations. The binding constants *K*_1_^*t*^ (eq 1) and *K*_2_^*t*^ (eq 3) corresponding to the binding of the first and second CP guests by the WP5 host, respectively, were determined by nonlinear least-squares curve fitting to the chemical shift changes of protons B and 1 (Figure S8). For *K*_1_^*t*^, a value of (3.2 ± 0.3) × 10^3^ M^–1^ was obtained, which is in good agreement with the binding constant calculated from the fluorescence spectra. For the binding of the second guest, *K*_2_^*t*^ = (8 ± 3) × 10^2^ M^–1^ was obtained. Thus, the two binding constants are related as *K*_1_^*t*^ = 4*K*_2_^*t*^, which is characteristic of noncooperative 1:2 systems, where the two binding sites are energetically identical.^51^

A set of the spectra of *trans*-CP measured at different WP6 concentrations is displayed in Figure S6. The molar ratio plots of the chemical shifts (Figure S10) confirm the 1:1 stoichiometry of the complex.

We note that on the addition of WP5 or WP6 in low concentrations, the solution of *trans*-CP became turbid, indicating the formation of aggregates, and from WP5/WP6 concentrations of 0.2 equiv, the samples were clear. Aggregation frequently occurs in mixtures of multiply charged macrocyclic hosts with oppositely charged guests when the latter is in excess.^72^ The formation of aggregates at the concentrations used in the NMR titrations was confirmed by the absorption spectra of mixtures with similar compositions. The transmittance values of *trans*-CP–WP5 and *trans*-CP–WP6 mixtures at 800 nm where the absorption of CP and its WP5/WP6 complexes are negligible, reached a minimum at [WP5]/[*trans*-CP] and [WP6]/[*trans*-CP] ratios of ≈0.1. Similar experiments in the concentration ranges of the photometric and fluorometric titrations indicated no aggregation.

### 3.5 Theoretical Calculations

**Figure 8:**
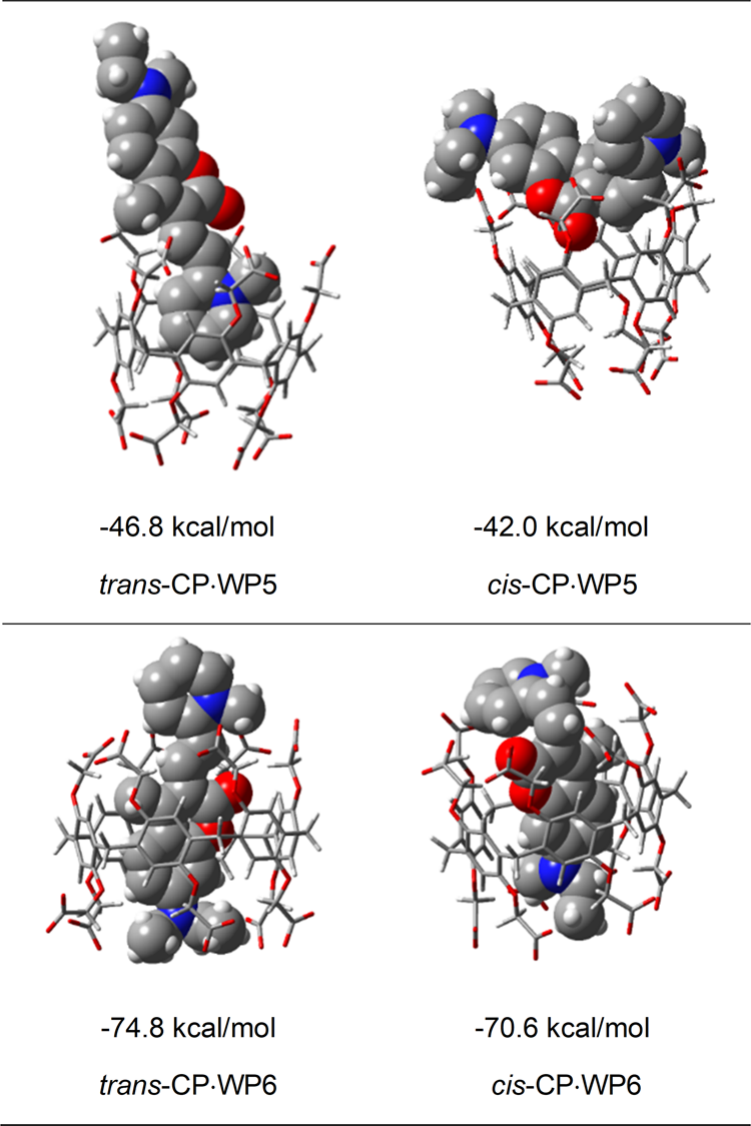
Theoretically calculated structures and binding energies of the CP·WP5 and CP·WP6 complexes.

Theoretical calculations have provided descriptive images of the modes of binding of the photoisomers of CP to the pillararene hosts. In addition, the experimental values of the binding constants, determined by spectroscopic methods, could be interpreted on the basis of these calculations. The optimized structures and stabilization energies of the *trans*-CP·WP5 and *cis*-CP·WP5 complexes are displayed in Figure 8.

As can be seen in the figure, the complexes formed by *trans*-CP as well as *cis*-CP with WP5 are essentially external complexes, although the pyridinium ring of *trans*-CP intrudes to some extent into the aromatic cavity of WP5. In a further possible structure for *trans*-CP·WP5, the diethylamino side of CP is oriented toward the cavity of the WP5 host (see Figure S12). This structure is, however, less stable than the one with the pyridinium unit of CP encapsulated in the WP5 host. The complexes are stabilized primarily by the electrostatic interaction between the oppositely charged carboxylate groups of the host and the pyridinium or diethylamino group of the guest; presumably, the latter group also possesses a partial positive charge.

The structures of the WP6 complexes of the two photoisomers are also displayed in Figure 8. These are both internal complexes held together by multiple interactions, such as π–π interactions, electrostatic interactions, and hydrophobic interactions. As indicated by the NMR spectra, the diethylamino-coumarin moiety is confined within the macrocyclic host in both complexes, whereas the pyridinium unit is located externally. In accordance with the experimental findings, these internal complexes formed with WP6 are much more stable than the external complexes formed with WP5, and the WP6 complex of *trans*-CP is somewhat more stable than the WP6 complex of the *cis* isomer.

Two possible arrangements for 2:1 complexes of the *trans* isomer with WP5 are exhibited in Figure S12. The structures differ on which functional group of the guest, the pyridinium or the diethylamino group, is coordinated by the carboxylate groups of the host. The binding energies of the 2:1 complexes are very close to the sums of the binding energies of the respective 1:1 complexes, confirming the noncooperative nature of the two-step binding process, indicated by the NMR experiments.

The results of our NMR experiments and theoretical calculations show that the complexes of the anionic WP5 and WP6 hosts with the cationic photoswitch CP show a variety of structures: they can form inclusion and exclusion complexes; in addition to the 1:1 complexes, 2:1 complexes have also been observed, and the CP guest can be oriented to the cavity of the pillararenes in different directions. XRD studies on crystalline complexes of WP5 and WP6 with various cationic guests (tetracaine,^73^ viologens,^74,75^ pentamidine,^76^ and biguanidium cations^77^) also demonstrated the structural diversity of these complexes and revealed that in some cases, these anionic hosts form different structures with the same cationic guests even in the crystalline state.

## 4 Conclusions

The water-soluble 7-diethylamino-coumarin derivative, CP, operates as a stilbene-type reversible molecular photoswitch. It has a fluorescent *trans* and a nonfluorescent *cis* isomeric form. The two isomers form much more stable complexes with the water-soluble pillararene WP6 than with its smaller homologue WP5. The large differences in the binding constants are consistent with the structural differences between WP5 and WP6 complexes. As was shown by NMR measurements and theoretical calculations, the two isomers of CP are encapsulated within the cavity of WP6, whereas they are coordinated only externally to WP5. In addition to the formation of 1:1 complexes, WP5 and WP6 with their two multicarboxylate coordination spheres can bind a second *trans*-CP guest. The binding of the two guests by WP5 is noncooperative, whereas WP6 binds the two guests with negative cooperativity. In terms of potential applications, the inclusion complexes of molecular photoswitch guests with macrocyclic hosts, such as the CP·WP6 adducts, attract much interest. The WP6 complex of the planar *trans*-CP is more stable than the complex of the bent *cis* isomer, the difference in the binding constants is, however, relatively small, allowing the repetitive switching between the *trans* and *cis* isomeric forms of the complexed photoswitch. Closed in the cavity of WP6, the deactivation of the excited CP molecules via the TICT state is suppressed. This results in the enhancement of the fluorescence quantum yield of the *trans* isomer and the enhancement of the photoisomerization quantum yield both in the *trans*-to-*cis* and the *cis*-to-*trans* directions. These findings may be helpful in the design of supramolecular photoswitching systems for fluorescence imaging applications.

## Supporting Information Available

NMR spectra of photoswitch CP and its mixtures with pillararenes WP5 and WP6 (PDF)
