# Supplementary material for: Supramolecular Control of the Photoisomerization of a Coumarin-Based Photoswitch
Source: ACS Omega. 2024 Dec 17;9(52):51652–64. doi: 10.1021/acsomega.4c08106 (PMC11696389; doi:10.1021/acsomega.4c08106)
Supplement: Supplementary file 1 — ao4c08106_si_001.pdf [file ao4c08106_si_001.pdf]

Supporting Information

Supramolecular control of the photoisomerization of a coumarin based photoswitch

Etelka Kiss [a], Dávid Mester [a,b,c], Márton Bojtár [d], Zsombor Miskolczy [e], László Biczók [e], Dóra Hessz [a], Mihály Kállay [a,b,c] and Miklós Kubinyi [a,]*

[a] Department of Physical Chemistry and Materials Science, Faculty of Chemical Technology and Biotechnology, Budapest University of Technology and Economics, 1111, Budapest, Műegyetem rkp. 3, Hungary

[b] MTA-BME Lendület Quantum Chemistry Research Group, Budapest University of Technology and Economics, 1111, Budapest, Műegyetem rkp. 3, Hungary;

[c] ELKH-BME Quantum Chemistry Research Group, Budapest University of Technology and Economics, 1111, Budapest, Műegyetem rkp. 3, Hungary;

[d] Chemical Biology Research Group, Institute of Organic Chemistry, Research Centre for Natural Sciences, 1117, Budapest, Magyar Tudósok krt. 2, Hungary

[e] Institute of Materials and Environmental Chemistry, Research Centre for Natural Sciences, HUN-REN Research Network, H-1519 Budapest, P.O. Box 286, Hungary

Table of contents

S1. NMR spectra 2

S2. Photokinetic experiments 3

S3. Chemical shifts of the isomeric forms of CP in their WPn complexes 4

S4. NMR spectra of the PSS mixtures 5

S5. Determination of the stoichiometry of *trans*-CP-WPn complexes 6

S6. Theoretical calculations 10

[*] Corresponding author.
E-mail address: kubinyi.miklos@vbk.bme.hu (M. Kubinyi).

S1. NMR spectra

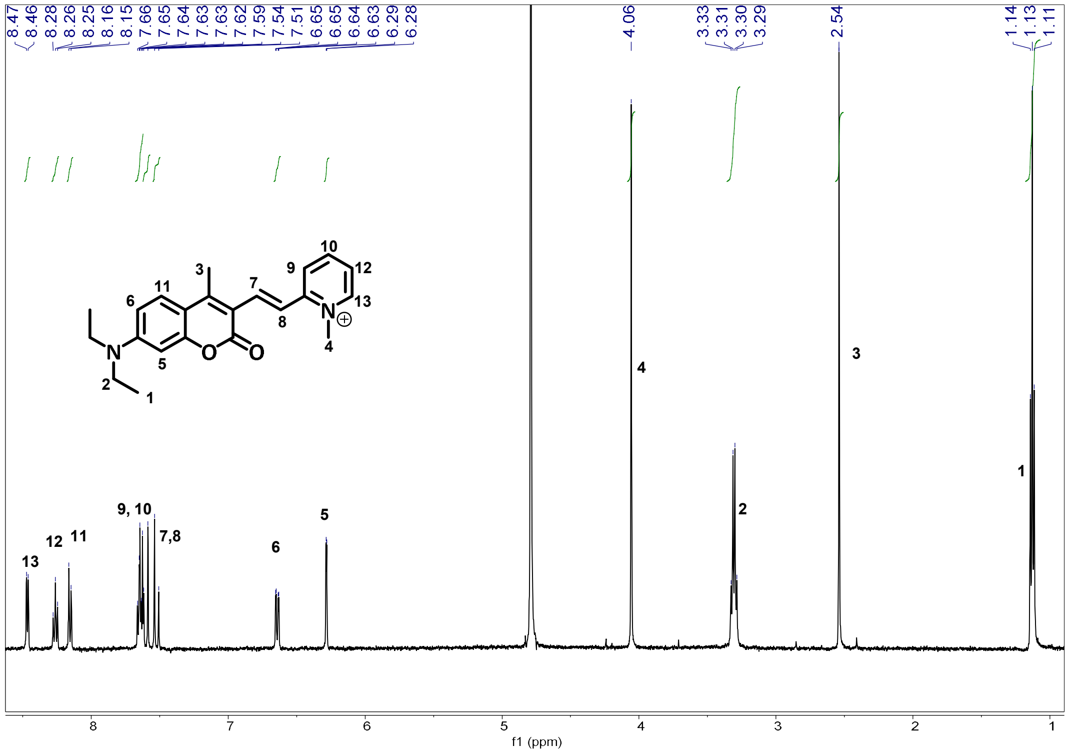

Fig. S1: ^{1}H-NMR spectrum of *trans*-CP at 500 MHz, D_2O.

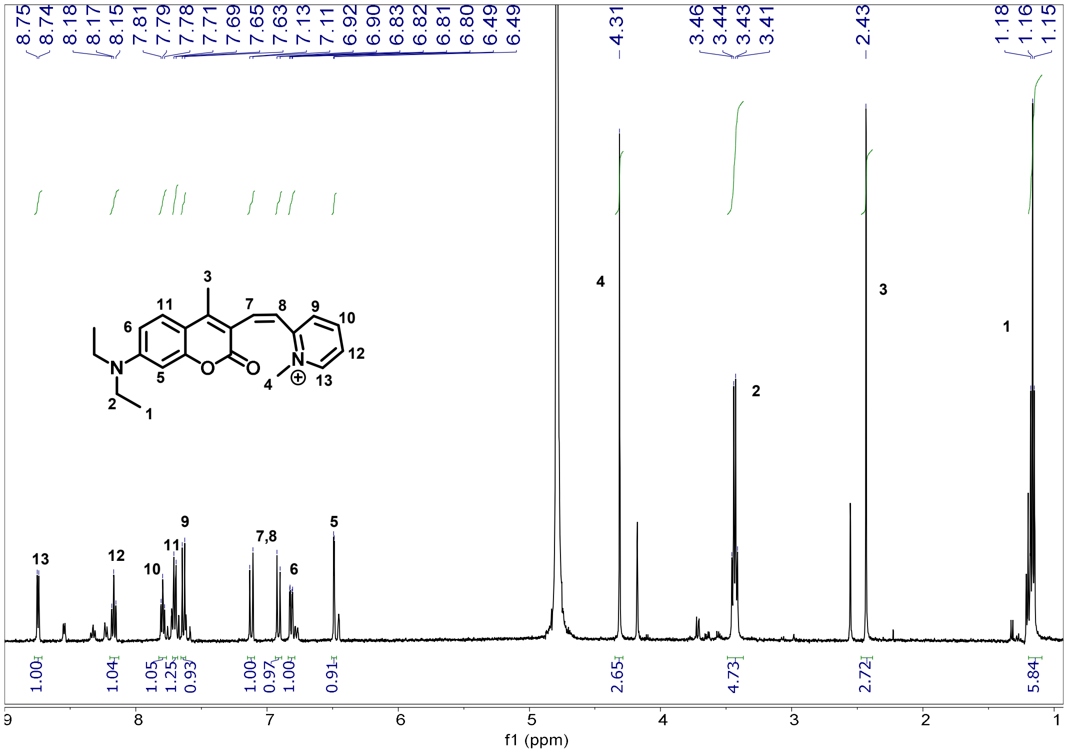

Fig. S2: ^{1}H-NMR spectrum of a PSS containing 75% *cis*-CP at 500 MHz in D_2O

S2. Photokinetic experiments

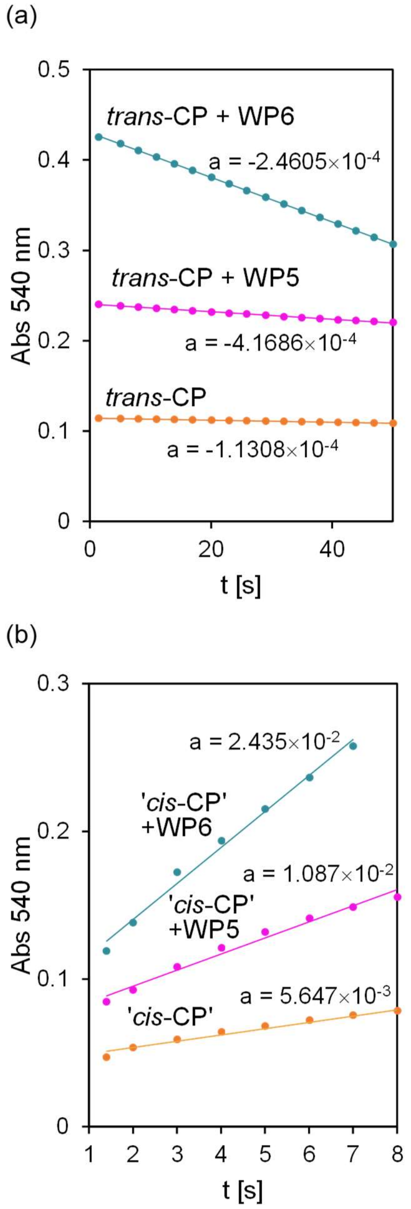

Fig. S3: Initial $A^D(t)$ data of (a) of *trans*-CP, and its mixture with WP5 and WP6, irradiated with the green (540 nm) LED; (b) a PSS system with 75% *cis*-CP and its mixtures with WP5 and WP6, irradiated with the UV (370 nm) LED. "a" is the slope of linearly fitted data (s^{-1}). Concentrations as given in the captions of Figs. 5 and 6.

S3. Chemical shifts of the isomeric forms of CP in their WPn complexes

Table S1: Chemical shifts of *trans*-CP, *cis*-CP and their WP5 complexes

	**WP5**	***trans*-CP**		**’*cis*-CP’**	
	free	*free*	*1 equiv. WP5*	*free*	*1 equiv. WP5*
**Proton**	*δ [ppm]*	*δ [ppm]*		*δ [ppm]*	
A	4.27		4.16		4.19
B	3.84		3.78		3.81
C	6.73		6.64		6.67
1		1.20	1.09	1.16	0.97
2		3.55	3.23	3.43	3.21
3		2.55	2.39	2.43	2.36
4		4.18	3.95	4.31	4.25
5		6.28	6.15	6.49	6.35
6		6.64	6.51	6.82	6.73
7		7.52	7.38	6.91	6.83
8		7.60	7.53 – 7.43	7.12	7.03
9		7.68 – 7.62	7.53 – 7.43	7.70	7.64
10		7.68 – 7.62	7.53 – 7.43	7.79	7.70
11		8.16	8.05	7.64	7.56 – 7.46
12		8.27	8.10	8.17	8.04
13		8.47	8.31	8.75	8.66

Table S2: Chemical shifts of *trans*-CP, *cis*-CP and their WP6 complexes

	**WP6**	***trans*-CP**		***cis*-CP**	
	free	*free*	*1 equiv. WP6*	*free*	*1 equiv. WP6*
**Proton**	*δ [ppm]*	*δ [ppm]*		*δ [ppm]*	
A	4.07		4.23		4.27
B	3.86		3.86		3.87
C	6.64		6.79		6.82
1		1.20	0.16	1.16	– 0.15
2		3.55	2.16	3.43	1.88
3		2.55	2.49	2.43	2.24
4		4.18	4.05	4.31	4.25 – 4.09
5		6.28	5.58	6.49	5.61
6		6.64	?	6.82	?
7/8		7.52	7.09	6.91	?
7/8		7.67 – 7.61	7.62 – 7.42	7.12	7.13
9		7.67 – 7.61	7.79 – 7.42	7.70	7.78 – 7.50
10		7.67 – 7.61	7.79 – 7.42	7.79	7.78 – 7.50
11		8.16	8.16 – 8.02	7.64	7.78 – 7.50
12		8.27	8.16 – 8.02	8.17	8.08
13		8.47	8.33	8.75	8.70

S4. NMR spectra of the PSS mixtures

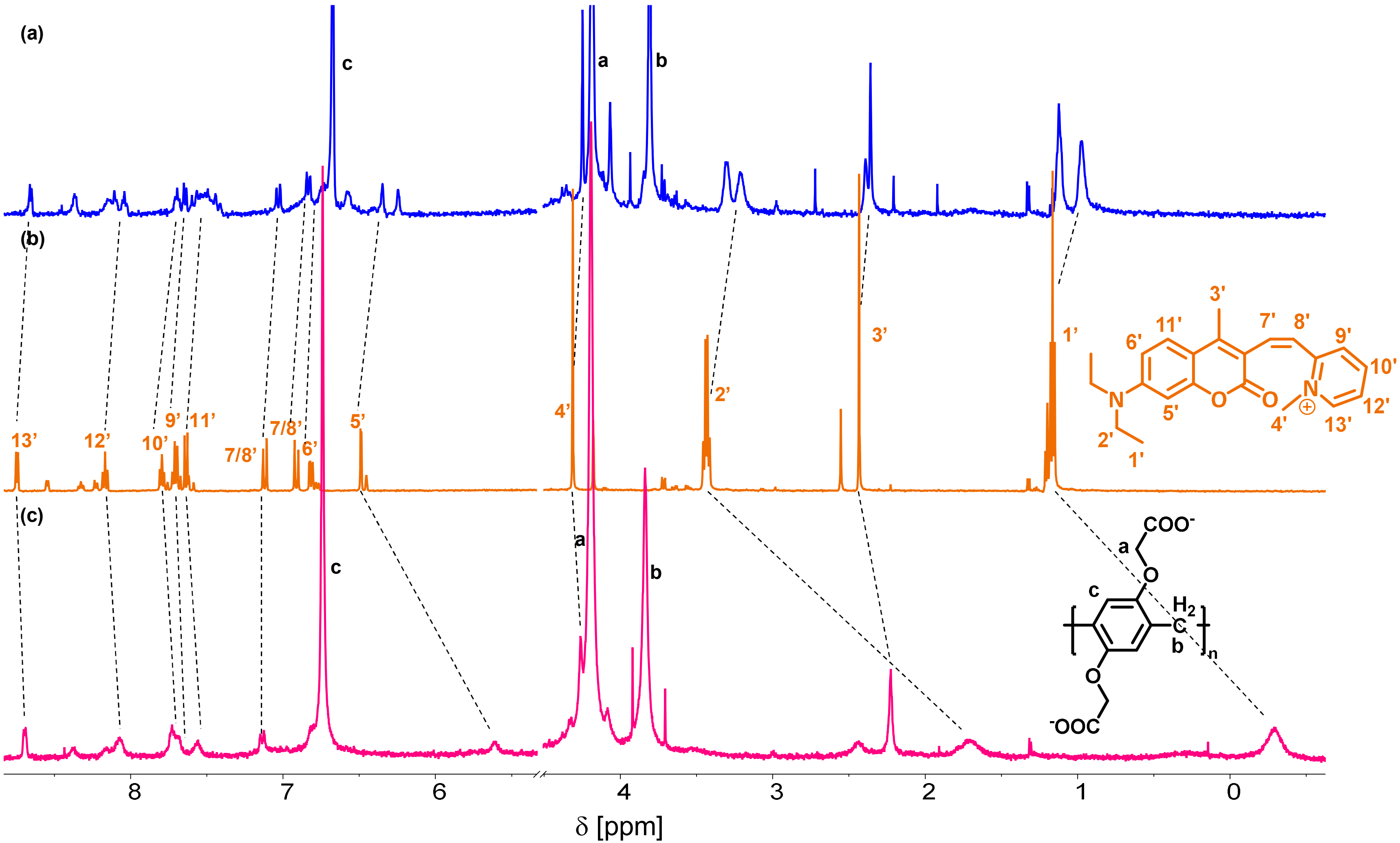

Fig. S4: (a) *trans*-CP + WP5 after 10 min irradiation with the green LED, (b) a *cis*-CP/*trans*-CP PSS mixture containing 75% *cis*-CP, (c) *trans*-CP + WP6 after 10 min irradiation with the green LED at 500 MHz, D_2O; [CP] = 6×10^{-4} M.

S5. Determination of the stoichiometry of the *trans*-CP-WPn complexes

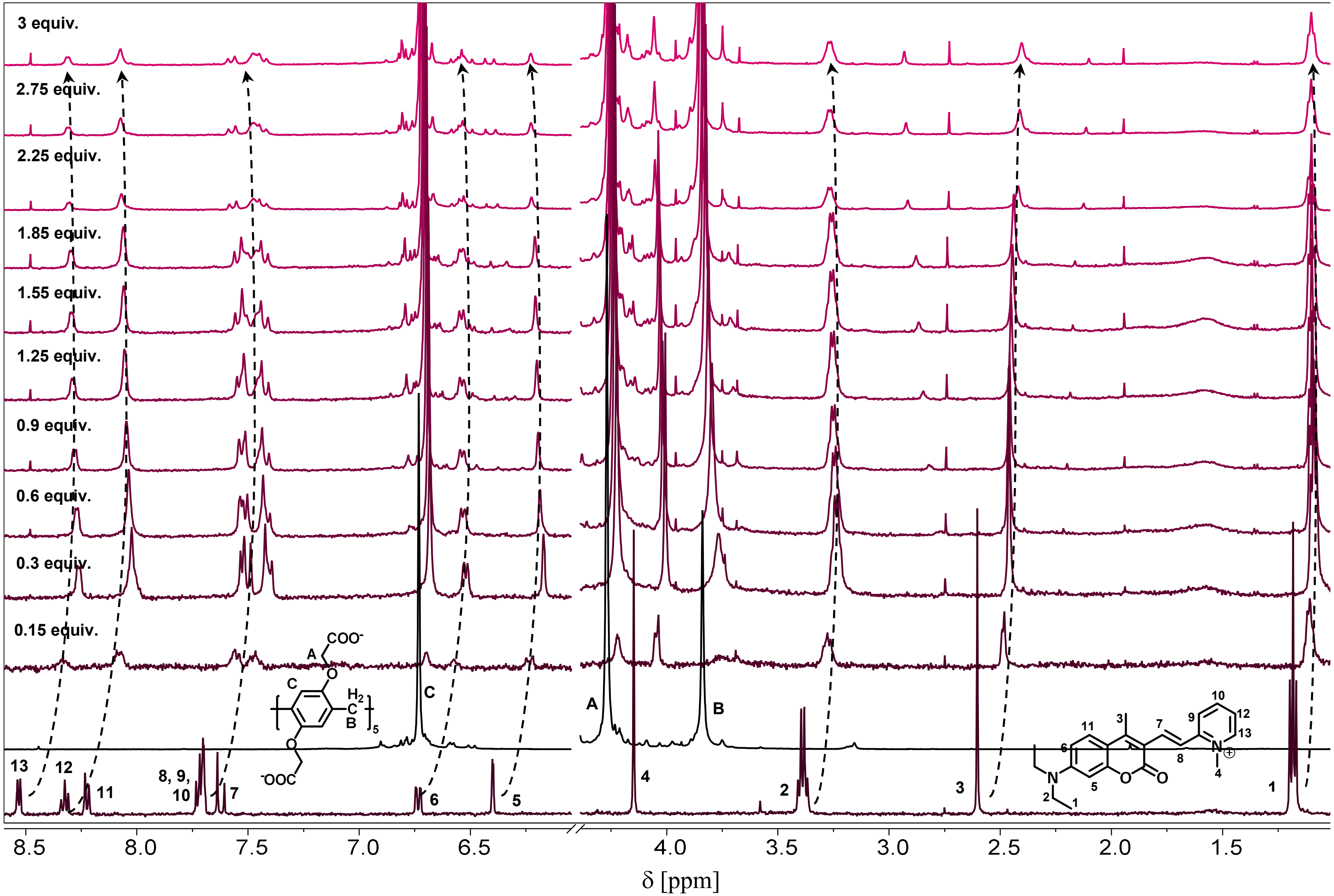

Fig. S5: ^{1}H-NMR titration of *trans*-CP in D_2O containing 0 – 3 equiv. of WP5, [CP] = 8×10^{-4} M

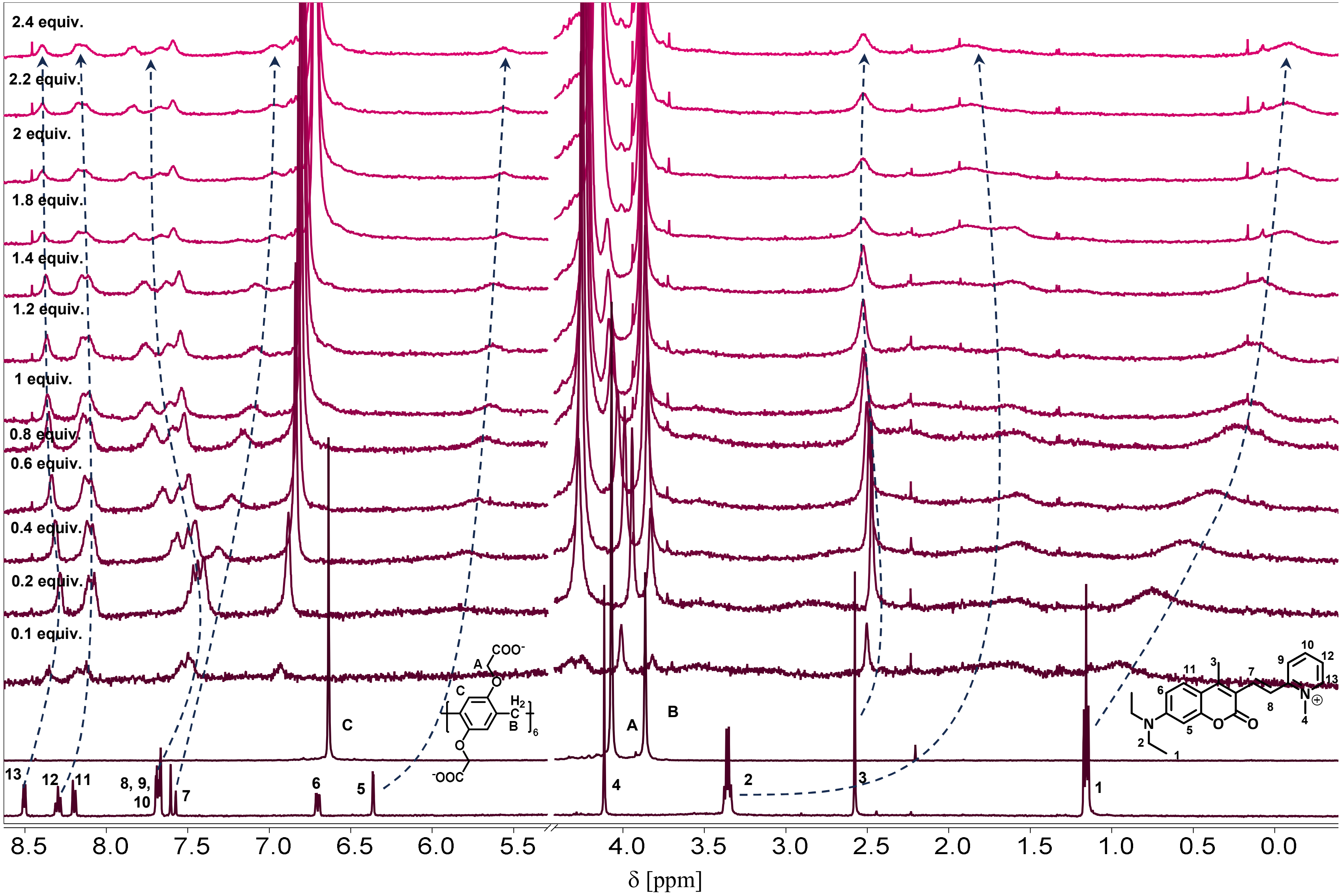

Fig. S6: ^{1}H-NMR titration of *trans*-CP in D_2O containing 0 – 2.4 equiv. of WP6, [CP] = 7×10^{-4} M

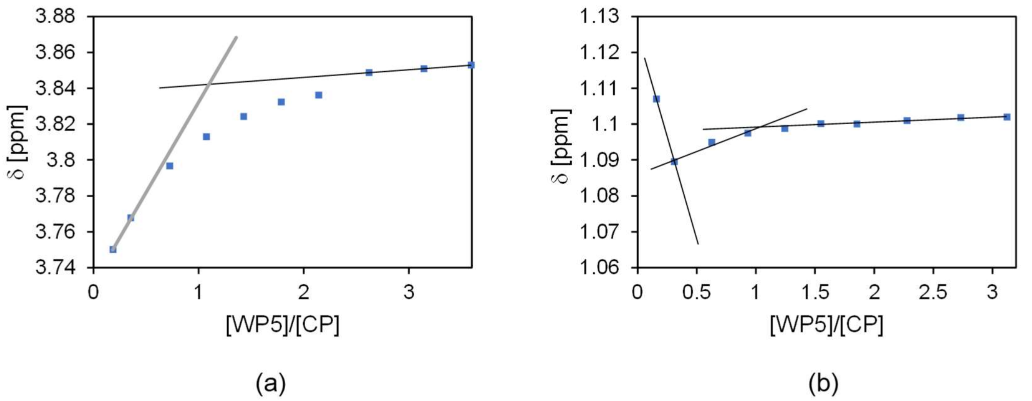

Fig. S7: Molar ratio plot for (a) **B** and (b) **1** protons in *trans*-CP·WP5-complex, [CP] = 8×10^{-4} M

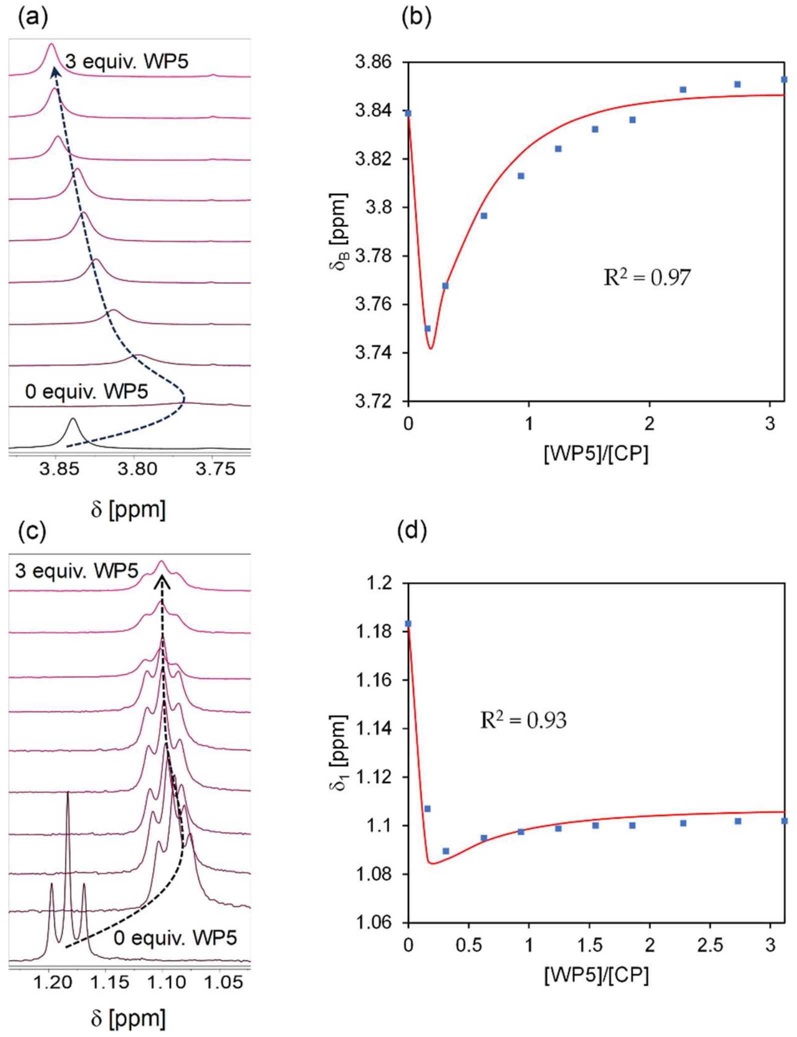

Fig. S8: Ranges of the ^{1}H NMR spectra (500 MHz, D_2O) presenting protons **B** and **1** in *trans*-CP and 0 – 3 equiv. WP5 mixtures (a and c); binding constant least-squares fitting for **B** (b) and **1** (d) protons in *trans*-CP complex

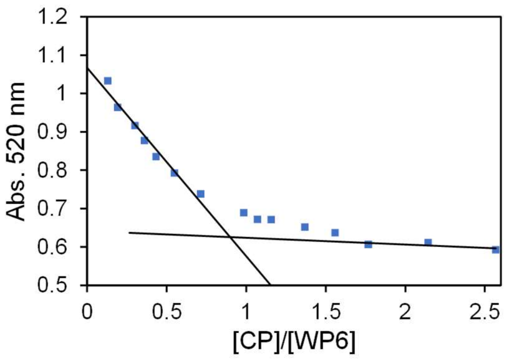

Fig. S9: Molar ratio plot for the *trans*-CP·WP6 complex, [CP] = 4×10^{-5} M

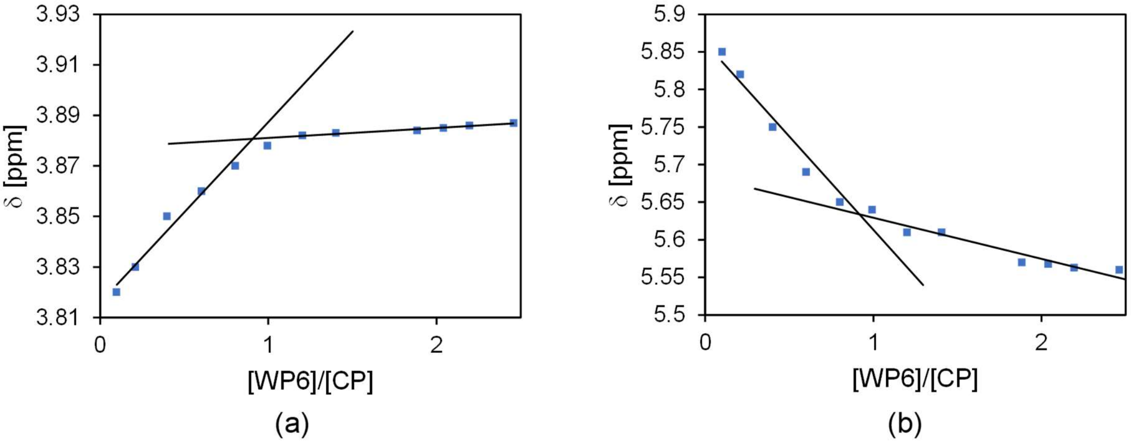

Fig. S10: Molar ratio plot for (a) **B** and (b) **5** protons in the *trans*-CP·WP6 complex, [CP] = 7×10^{-4} M

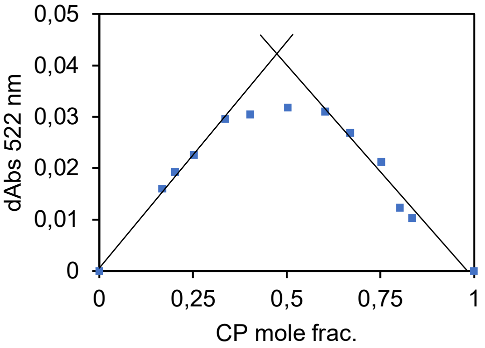

Fig. S11: Job's plot for determination of the stoichiometry of the *trans*-CP·WP6 complex; the sum of the concentrations of *trans*-CP and WP6 is $2\cdot10^{-5}$ M;

S6. Theoretical calculations

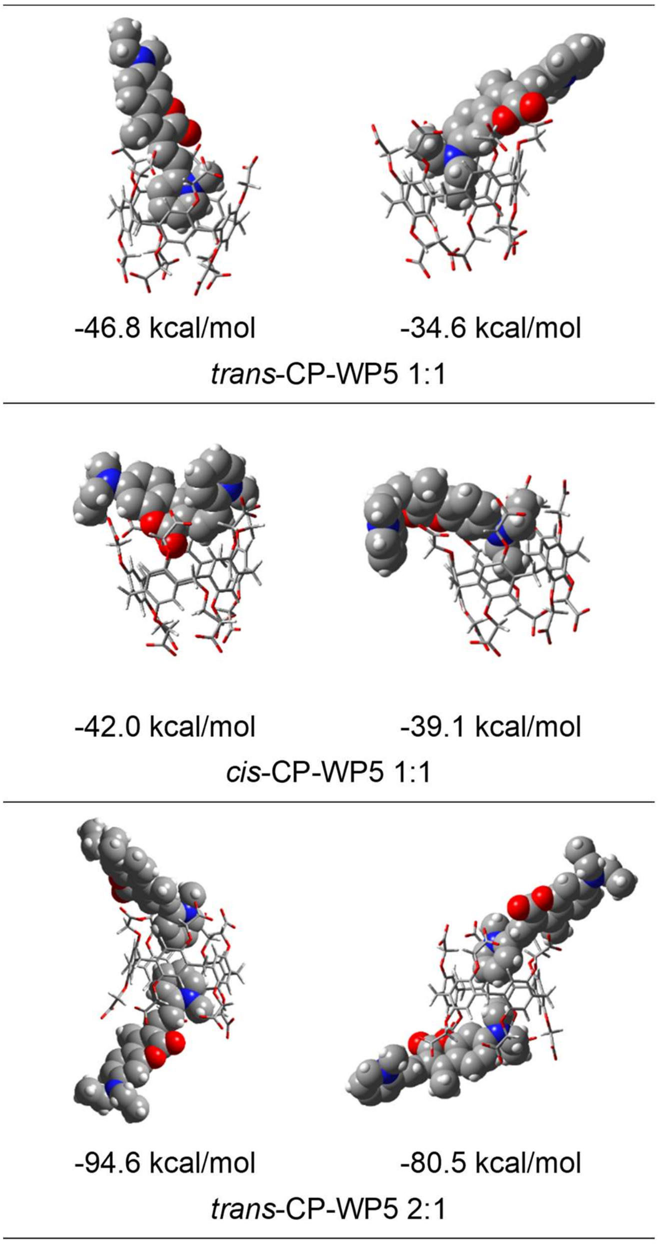

Fig. S12. Theoretically calculated structures and binding energies of CP-WP5 1:1 and 2:1 complexes
